# A comparison of blood flow restriction devices to assess limb occlusion pressure in supine and standing positions

**DOI:** 10.3389/fspor.2025.1654522

**Published:** 2025-11-04

**Authors:** Tulasiram Bommasamudram, Kirtana Raghurama Nayak, Matthew J. Clarkson, Rajagopal Kadavigere, Aaron P. Russell, Stuart A. Warmington

**Affiliations:** ^1^ Department of Exercise and Sports Sciences, Manipal College of Health Professions, Manipal Academy of Higher Education, Manipal, Karnataka, India; ^2^ Institute for Physical Activity and Nutrition (IPAN), School of Exercise and Nutrition Sciences, Deakin University, Geelong, VIC, Australia; ^3^ Department of Physiology, Kasturba Medical College, Manipal Academy of Higher Education, Manipal, Karnataka, India; ^4^ Department of Medical Education, Kasturba Medical College, Manipal Academy of Higher Education, Manipal, Karnataka, India; ^5^ Institute for Health and Sport, Victoria University, Melbourne, VIC, Australia; ^6^ Department of Radiodiagnosis and Imaging, Kasturba Medical College (KMC), Manipal Academy of Higher Education, Manipal, Karnataka, India

**Keywords:** Blood Flow Restriction, limb occlusion pressure, kaatsu, arterial occlusion pressure, occlusion

## Abstract

**Objective:**

The objective of this study was to compare five commercially available blood flow restriction (BFR) devices in determining limb occlusion pressure (LOP), plus two algorithm approaches for determining LOP, in both supine and standing positions.

**Methods:**

Twenty-one recreationally active males were assessed for LOP using five BFR devices: Zimmer (surgical-grade tourniquet; reference standard), AirBands, blood pressure cuff with pulse oximeter (BPPO), Smart Cuffs, and Suji. Two additional algorithms based on resting anthropometric/physiological data were also assessed. LOP was measured in both supine and standing positions, with two measurements per posture separated by a five-minute interval. In addition to LOP, participants rated their level of discomfort during each measurement.

**Results:**

When compared to the Zimmer device, BPPO (*r* = 0.636, *p* = 0.002) and Smart Cuffs (*r* = 0.758, *p* < 0.001) demonstrated the closest association in the supine and standing positions, respectively. AirBands exhibited the greatest deviation from Zimmer in both positions but were consistently rated as more comfortable (*p* > 0.05), even at higher pressures.

**Conclusion:**

None of the devices showed consistent LOP measurements across both postures, indicating significant variability depending on device type and body position. These findings underscore the need for posture-specific calibration when using BFR devices and caution against assuming device interchangeability.

## Introduction

Blood Flow Restriction (BFR) exercise utilises the application of a tourniquet cuff to a limb to restrict blood flow during physical activity (1). When applied at rest, BFR typically results in partial restriction of arterial inflow and full occlusion of venous outflow (2). When combined with low-intensity exercise, BFR produces physiological responses and training adaptations that are greater than those seen with intensity-matched exercise without BFR, while at times being comparable to adaptations observed with high-intensity training (3–6). These adaptations include increases in muscle size, muscle strength, and aerobic capacity (3). BFR training is suited for use in populations where high-intensity training is not possible due to injury, illness, or other limitations, as it allows for improvements in physical function with lower mechanical stress on joints and connective tissues (7).

Initial approaches to prescribe BFR pressures employed absolute cuff pressures without reference to an individual's characteristics (8) or determined lower-limb pressures as a proportion of brachial systolic blood pressure (9, 10). These strategies did not account for individual differences such as limb circumference, cuff width, or vascular responsiveness (11). Currently, individualized approaches are proposed to improve both safety and effectiveness of BFR training (11), with measurement of limb occlusion pressure (LOP) emerging as the primary method for tailoring BFR pressure to the individual. Current recommendations suggest using BFR pressures between 40% and 80% of LOP (12).

A variety of devices are now commercially available for estimating LOP. These range from manual tools like sphygmomanometers used in conjunction with Doppler ultrasound, to fully automated pneumatic systems (13, 14). These devices differ significantly in their design, operational characteristics (e.g., autoregulating vs. non-autoregulating), and cost (15–17). This can complicate the process for selecting appropriate devices for both practitioners and researchers (16). Importantly, not all devices are equally accurate or reliable. For instance, Keller et al. (18) demonstrated that while the AirBands device provided reasonable LOP estimates in the upper limbs, it consistently reached a ceiling threshold of 270 mmHg in the lower limbs, suggesting it may not be suitable for leg applications. This highlights a broader concern: variability in LOP measurement across devices can influence both the safety and effectiveness of BFR training (11). Beyond device-related differences, algorithm-based methods that incorporate physical and physiological variables (e.g., limb circumference, resting blood pressure) have been proposed to estimate LOP, offering an alternative to direct measurement [Tuncali et al. (19) and Loenneke et al. (20)]. Moreover, despite the growing availability of portable and affordable LOP devices has facilitated the use of BFR outside clinical or laboratory settings, their accuracy across different postures remains uncertain (16).

Therefore, the objective of this study is to compare LOP values obtained from several commercially available BFR devices with those from a surgical-grade tourniquet system, and to determine the influence of posture (supine vs. standing) on these measurements. Given the range of equipment being tested and the prior demonstration of variable outcomes from emerging BFR devices, we hypothesize that the range of commercially available BFR devices examined will systematically over-/under-estimate LOP when compared with a surgical-grade tourniquet system, produce diversity in their accuracy, reliability and acceptability for use in different postures, while we expect LOP to be higher in the standing position when compared with the supine position.

## Methodology

### Participants

Twenty-one recreationally active males volunteered to participate in this study (mean age: 22.7 ± 3.7 years; height: 175.5 ± 6.3 cm; body mass: 73.5 ± 11.8 kg). All participants were right-leg dominant, with an average mid-thigh circumference of 55.3 ± 2.7 cm (Table 1). Participants were recruited via convenience sampling and contacted the research team to express interest. Screening assessments were conducted in the laboratory and included anthropometric measurements, resting blood pressure. Individuals were excluded if they reported any known cardiovascular, metabolic, or musculoskeletal conditions. All participants received detailed verbal and written information outlining the study's procedures, risks, and benefits before providing written informed consent. The study was approved by the Human Ethics Advisory Group of Deakin University (HEAG-H 105_2024), registered in the Australian New Zealand Clinical Trials Registry (ACTRN12625000398404), and conducted in accordance with the Declaration of Helsinki.

**Table 1:** Baseline characteristics of the participants.

Participants characteristics	
Age (years)	22.7 ± 3.7
Height (cm)	175.5 ± 6.3
Weight (kg)	73.5 ± 11.8
BMI (kg.m^−2^)	23.8 ± 3.1
Mid-thigh circumference (cm)	55.3 ± 2.7
Resting systolic blood pressure (mmHg)	120.1 ± 9.3
Resting diastolic blood pressure (mmHg)	72.5 ± 7.1

### Study design

This was a single-session, within-subject observational study designed to compare limb occlusion pressure (LOP) measurements across five different blood flow restriction (BFR) devices in two body positions (supine and standing). The five devices included a gold-standard surgical-grade device (Zimmer A.T.S.® 4000 Tourniquet System). In addition to these devices, we included two validated algorithms which estimate LOP based on resting systolic blood pressure and thigh circumference (19, 20).

Upon arrival, participants underwent initial screening, which included measurement of height and body mass using a calibrated stadiometer and digital scale, and resting blood pressure using a standard sphygmomanometer after 5 min of seated rest. Participants were instructed to wear shorts and abstain from caffeine and food for ≥2 h, and from physical activity for ≥24 h prior to testing. All procedures were conducted in a temperature-controlled laboratory (21–24 °C).

Each participant completed a single laboratory session for 120–150 min, during which LOP was measured using all five BFR devices. The order of device testing was randomized using an online tool (Research Randomizer). For each device, LOP was assessed in both the supine and standing positions, always in that order. Two measurements were obtained per position. We set 15% as an *a priori* criterion to ensure consistency between repeated measurements. This equated to approximately 30 mmHg, slightly above the typical 20 mmHg used in our laboratory (21–23) in order to account for an expected increase in variability with non-surgical devices (18). If the two values differed by more than 15%, a third measurement was taken. When a third measurement was required, the two closest values were averaged and used for analysis. A 5-minute rest period was provided between devices and between positions to minimize carryover effects. This repeated-measures design yielded a minimum of ten LOP measurements per participant (5 devices × 2 positions). The independent variables were the BFR device and position, and the dependent variable was LOP (mmHg). In addition, participants rated their discomfort using a previously published Rating of Discomfort (ROD) scale ranging from 0 (no discomfort/pain) to 10 (extreme discomfort/unbearably painful) (24–26). ROD was recorded after each LOP measurement in both supine and standing positions.

### BFR devices

Five commercially available BFR devices were evaluated: (1) Zimmer (A.T.S.® 4000 Tourniquet System, USA; 10.5 cm wide), (2) AirBands (VALD Performance, Australia; 10.5 cm wide), (3) Smart Cuffs (Smart Tools, Ohio, USA; 10.5 cm wide), (4) Suji (Suji, Scotland, UK; 10 cm wide), and (5) blood pressure cuff with pulse oximeter [(BPPO); 15 cm wide].

Participants wore shorts to allow for cuff placement on the proximal thigh. LOP was assessed according to each manufacturer's instructions, except for BPPO. For the BPPO method, a 15 cm-wide blood pressure cuff (BCS080 Cuff Assembly, Obese) was applied to the thigh, and a pulse oximeter was attached to the participant's second toe. The cuff was inflated until the pulse oximeter no longer detected a pulse, indicating the LOP (4).

Additionally, we applied equation-based algorithms from Tuncali et al. (19) and Loenneke et al. (20), which utilize resting systolic and diastolic blood pressures along with thigh circumference, and compared the estimated values to those obtained from the Zimmer device in the standing position.

To enable pressure monitoring in the Suji, Smart Cuffs, and BPPO device, a T-connector was added to each cuff's tubing to facilitate connection to a custom pressure acquisition module. This module incorporated an MPX5100 pressure transducer and interfaced with the Better Serial Plotter software via USB, similar to prior investigations of multiple cuff devices/systems (13). The module contained no active components that could interfere with cuff operation or pressure delivery. No physical modifications were made to the Zimmer or AirBands devices.

### Measurement postures

LOP was measured in both supine and standing positions for each BFR device. In the supine position, participants lie down on a surgical bed with their head up on a pillow. In the standing position, participants stood on a stepper box to facilitate proper placement of the pulse oximeter on the second toe of the right foot. In both positions, BFR cuffs were placed on the proximal thigh of the right leg.

### Data analysis

All statistical analyses were conducted using Jamovi Statistics (v2.5.3; Sydney, Australia) obtained from https://www.jamovi.org and figures were produced using GraphPad Prism (v10.0.0 for Windows, GraphPad Software, Boston, Massachusetts USA, http://www.graphpad.com). Demographic and anthropometric data were presented as mean ± standard deviation (SD). The Shapiro–Wilk test was used to assess the normality of the data distribution, with all continuous variables meeting the assumption of normality. A repeated measures analysis of variance (ANOVA) was employed to evaluate the effects of Device, Position, and their interaction, while one-way ANOVA was used to compare devices against algorithm-based LOP data in the standing position only. Mauchly's test was used to assess the sphericity, and when violated, Greenhouse–Geisser corrections were applied. A Tukey *Post-hoc* analyses were performed where applicable. To evaluate the agreement between the gold-standard device and each of the other BFR devices/algorithms, separate Bland–Altman plots were generated. In addition, Pearson's correlation coefficients were calculated to assess the linear relationship between each device and Zimmer. In line with the threshold for repeat assessments of LOP in our method, we set an acceptable threshold of ±30 mmHg to calculate a percentage score of measures within the acceptable range. Correlation strength was interpreted as large (*r* ≥ 0.5), moderate (*r* ≥ 0.3), or small (*r* ≥ 0.1), in accordance with established guidelines (27). The Intraclass Correlation Coefficient (ICC) was calculated to assess the reliability of LOP measurements from each device in comparison with Zimmer. Additionally, to assess the unit change in pressure relative to discomfort, the ratio of LOP to ROD was calculated and compared across devices relative to Zimmer. Statistical significance was set at *p* < 0.05.

## Results

### Limb occlusion pressure

**Figure 1:**
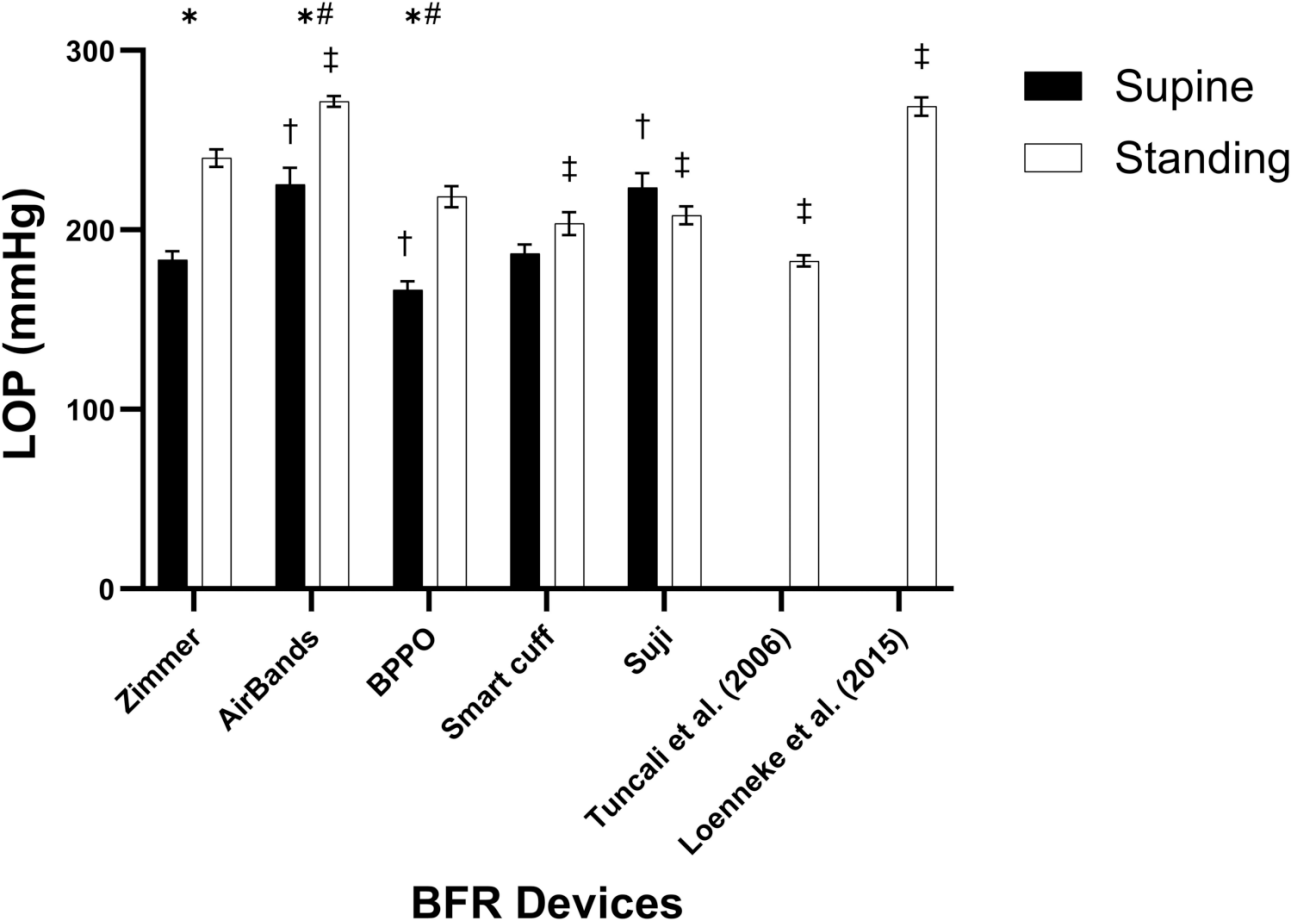
Limb occlusion pressure (LOP) measurements across different devices in supine and standing positions. * indicates a significant difference between the supine and standing positions within the same device (*p* < 0.05). # denotes a significant difference against the Zimmer device regardless of body position (*p* < 0.05). † indicates a significant difference against the Zimmer device in the supine position (*p* < 0.05), while the ‡ represents a significant difference against the Zimmer device in the standing position (*p* < 0.05). Values are presented as mean ± SEM.

There was a main effect for device (*F* = 23.0, *p* < 0.001, *η*^2^ = 0.240) such that when compared with Zimmer, the recorded LOP values for AirBands were higher, BPPO were lower, while Smart Cuffs and Suji were not different from Zimmer (Figure 1). There was also a main effect for posture (*F* = 112.0, *p* < 0.001, *η*^2^ = 0.163) such that LOP while standing was significantly higher than when supine.

However, given there was also a significant interaction (*F* = 16.9, *p* < 0.001, *η*^2^ = 0.119), *post-hoc* analysis revealed more specifically that when compared with Zimmer, significantly higher LOP recordings were observed while standing with AirBands, while being significantly lower with Smart Cuffs and Suji. Compared with Zimmer when supine, significantly higher LOP recordings were observed with AirBands and Suji, while being significantly lower with BPPO (Figure 1). In addition, LOP estimates based on the algorithms from Tuncali et al. (19) and Loenneke et al. (20) were observed to be significantly lower and higher, respectively, when compared with Zimmer.

### Validity, reliability & agreement

In the supine posture, the BPPO device demonstrated moderate reliability with an ICC of 0.67 (95% CI: −0.10 to 0.89) and a moderate Pearson correlation with the Zimmer (*r* = 0.636, *p* = 0.002). However, its bias (58.86 mmHg) and wide limits of agreement (LoA: −32.00 to 149.8 mmHg) indicate poor agreement. AirBands showed poor reliability (ICC = 0.26, 95% CI: −0.10 to 0.60) and moderate correlation (*r* = 0.547, *p* = 0.010), but also had high bias (41.98 mmHg) and wide LoA (–28.36 to 112.3 mmHg). Smart Cuffs exhibited poor reliability (ICC = –0.27, 95% CI: −2.40 to 0.50) and no meaningful correlation (*r* = –0.115, *p* = 0.619), with substantial bias (38.48 mmHg) and LoA (–71.6 to 148.5 mmHg). Suji showed poor reliability (ICC = –0.21, 95% CI: −0.45 to 0.20) and weak negative correlation (*r* = –0.435, *p* = 0.049), despite low bias (1.76 mmHg); its LoA (–109.5 to 113.1 mmHg) were the widest among all devices (Table 2; Supplementary Figure S1).

**Table 2:** Agreement between devices and the reference standard for LOP measurement, presented as bias, limits of agreement (LoA), proportion of measurements within the predefined acceptable range, intraclass correlation coefficient (ICC) with 95% confidence intervals (CI), and Pearson's correlation coefficient (*r*) with corresponding *p*-values.

Device	Bias (mmHg)	Lower LoA (mmHg)	Upper LoA (mmHg)	In-range (%)	ICC	95% CI	Pearson *r*	Pearson *p*
Supine								
BPPO	58.86	−32	149.8	33	0.67	[−0.10, 0.89]	0.636	0.002
AirBands	41.98	−28.36	112.3	38	0.26	[−0.10, 0.60]	0.547	0.01
Smart Cuffs	38.48	−71.6	148.5	48	−0.27	[−2.40, 0.50]	−0.115	0.619
Suji	1.76	−109.5	113.1	33	−0.21	[−0.45, 0.20]	−0.435	0.049
Standing								
BPPO	20.71	−38.01	79.44	57	0.39	[−0.33, 0.74]	0.317	0.162
AirBands	−32.45	−75.07	10.17	48	0.26	[−0.30, 0.67]	0.411	0.064
Smart Cuffs	35.65	−1.14	72.45	38	0.57	[−0.23, 0.87]	−0.771	0.001
Suji	30.95	−23.31	85.21	43	0.27	[−0.37, 0.67]	0.295	0.195
Loenneke et al. (2015)	−29.65	−72.62	13.33	67	0.49	[−0.35, 0.80]	0.575	0.006
Tuncali et al. (2006)	56.43	13.59	99.27	9	0.13	[−0.13, 0.50]	0.412	0.063

In the standing posture, Smart Cuffs demonstrated moderate reliability (ICC = 0.57, 95% CI: −0.23 to 0.87) but a strong negative correlation with the Zimmer (*r* = –0.771, *p* < 0.001), suggesting inverse tracking. Its bias (35.65 mmHg) and LoA (–1.14 to 72.45 mmHg) remained outside acceptable limits. BPPO showed poor reliability (ICC = 0.39, 95% CI: −0.33 to 0.74) and weak correlation (*r* = 0.317, *p* = 0.162), with bias of 20.71 mmHg and LoA of −38.01 to 79.44 mmHg. AirBands had poor reliability (ICC = 0.26, 95% CI: −0.30 to 0.67) and moderate correlation (*r* = 0.411, *p* = 0.064), but bias (–32.45 mmHg) and LoA (–75.07 to 10.17 mmHg) exceeded acceptable thresholds. Suji showed poor reliability (ICC = 0.27, 95% CI: −0.37 to 0.67) and weak negative correlation (*r* = –0.295, *p* = 0.195), with bias of 30.95 mmHg and LoA of −23.31 to 85.21 mmHg (Table 2; Supplementary Figure S2).

Algorithm-based estimates also demonstrated poor reliability. Tuncali et al. showed an ICC of 0.13 (95% CI: −0.13 to 0.50) and moderate correlation (*r* = 0.412, *p* = 0.063), with bias of 56.43 mmHg and LoA of 13.59 to 99.27 mmHg. Loenneke et al. had borderline moderate reliability (ICC = 0.49, 95% CI: −0.35 to 0.80) and moderate correlation (*r* = 0.575, *p* = 0.006), with bias of −29.65 mmHg and LoA of −72.62 to 13.33 mmHg (Table 2; Supplementary Figure S3).

While no universal threshold exists for the percentage of measurements within the acceptable range, values above 80%–90% are generally considered indicative of strong agreement. In this study, all devices fell below this threshold with a range of 33%–48% in the supine posture and 9%–67% while standing. Only the Loenneke et al. algorithm achieved a respectable 67% (standing), with all other measures below 50%, thus reinforcing a general interpretation of poor agreement and reliability.

### Rating of discomfort (ROD)

**Figure 2:**
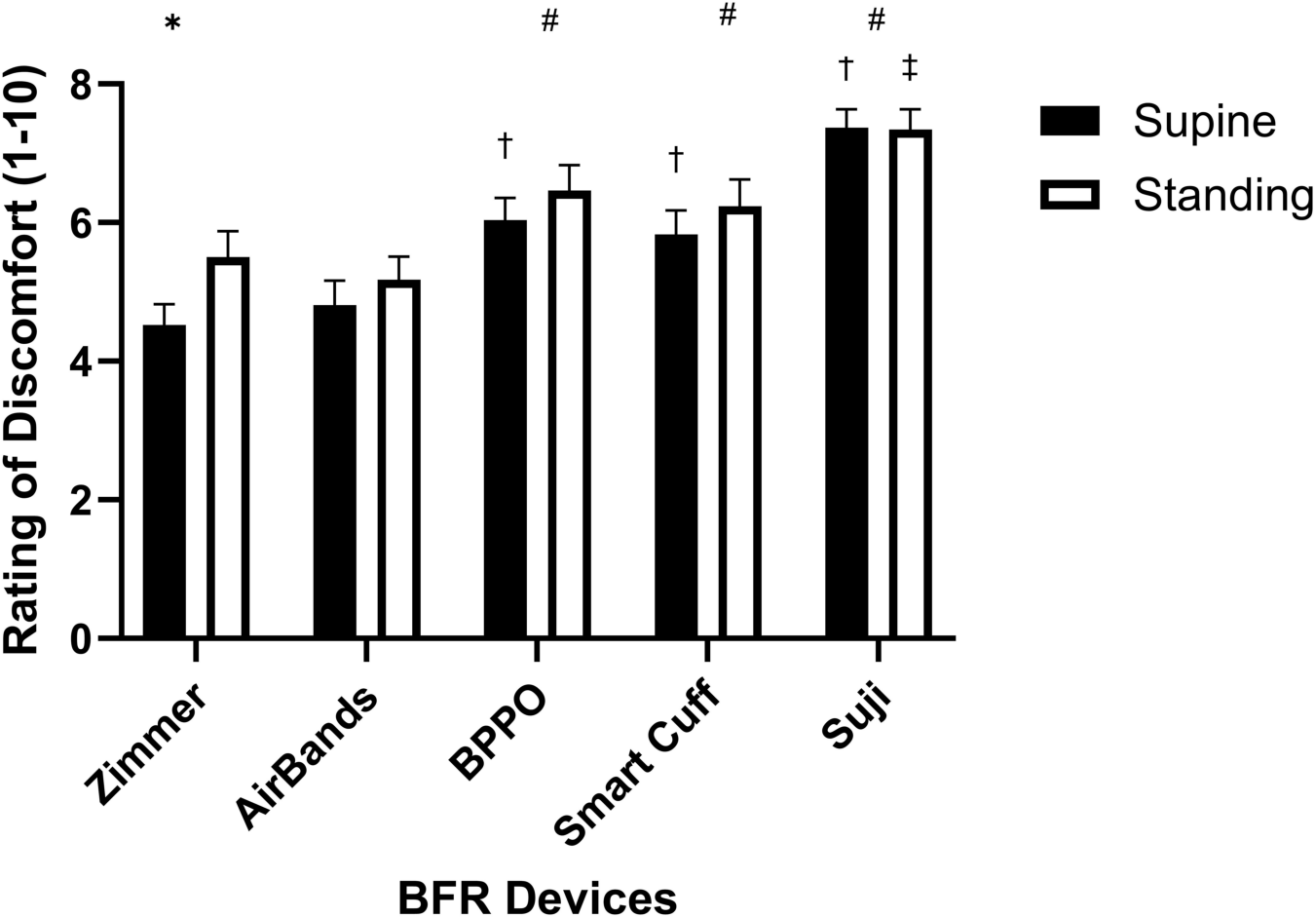
Rating of discomfort (ROD) measurements across different devices in supine and standing positions. * indicates a significant difference between the supine and standing positions within the same device (*p* < 0.05). # denotes a significant difference against the Zimmer device regardless of body position (*p* < 0.05). † indicates a significant difference against the Zimmer device in the supine position (*p* < 0.05), while the ‡ represents a significant difference against the Zimmer device in the standing position (*p* < 0.05). Values are presented as mean ± SEM.

There was a main effect for device (*F* = 40.62, *p* < 0.001, *η*^2^ = 0.241) such that when compared with Zimmer, the recorded ROD values for AirBands, BPPO, Smart Cuffs, and Suji were higher (Figure 2). There was also a main effect for posture (*F* = 9.75, *p* = 0.003, *η*^2^ = 0.014) such that ROD while standing was significantly higher than when supine.

However, given there was also a significant interaction (*F* = 1.335, *p* = 0.011, *η*^2^ = 0.008), *post-hoc* analysis revealed more specifically that when compared with Zimmer, significantly higher ROD recordings were observed while standing with Suji, while there was no difference vs. AirBands, BPPO, and Smart Cuffs. Compared with Zimmer when supine, significantly higher ROD recordings were observed with BPPO, Smart Cuffs, and Suji while there was no difference vs. AirBands (Figure 2).

### Pressure-to-discomfort ratio (LOP.ROD^−1^)

**Figure 3:**
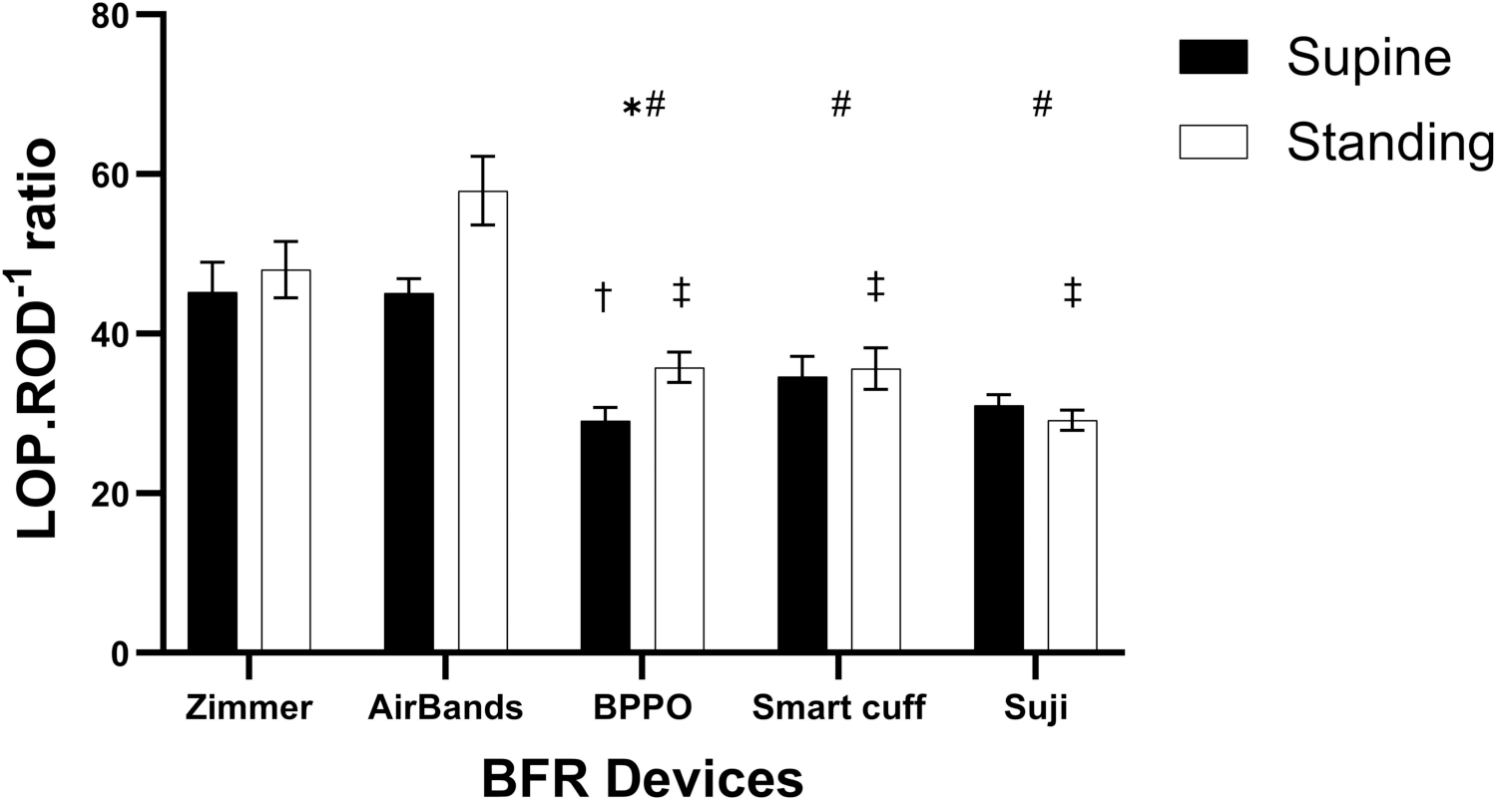
The ratio of limb occlusion pressure to rating of discomfort (LOP.ROD^−1^) across different devices in supine and standing positions. * indicates a significant difference between the supine and standing positions within the same device (*p* < 0.05). # denotes a significant difference against the Zimmer device regardless of body position (*p* < 0.05). † indicates a significant difference against the Zimmer device in the supine position (*p* < 0.05), while the ‡ represents a significant difference against the Zimmer device in the standing position (*p* < 0.05). Values are presented as mean ± SEM.

There was a main effect for device (*F* = 29.39, *p* < 0.001, *η*^2^ = 0.304) such that when compared with Zimmer, the LOP.ROD^−1^ ratio was significantly lower for BPPO, Smart Cuffs, and Suji except for AirBands which was no different (Figure 3). There was also a main effect for posture (*F* = 10.75, *p* = 0.004, *η*^2^ = 0.028) such that the LOP.ROD^−1^ ratio while standing was significantly higher than when supine.

However, given there was also a significant interaction (*F* = 4.31, *p* = 0.0031, *η*^2^ = 0.028), *post-hoc* analysis revealed more specifically that when compared with Zimmer, the LOP.ROD^−1^ ratio while standing was significantly lower with BPPO, Smart cuffs, and Suji, while there was no difference vs. AirBands. Compared with Zimmer when supine, the LOP.ROD^−1^ ratio was also significantly lower with BPPO, while there was no difference vs. AirBands, Smart Cuffs, and Suji (Figure 3).

## Discussion

The present study aimed to compare LOP measurements taken in both supine and standing positions using various commercially available BFR devices, and to compare these against a surgical-grade tourniquet as the reference standard. The results reveal variability in LOP measures between devices and positions. In the supine position, BPPO significantly underestimated LOP by 9% compared to Zimmer, while AirBands and Suji significantly overestimated LOP by 23% and 22%, respectively. Smart Cuffs displayed comparable performance to Zimmer. Conversely, in the standing position, AirBands significantly overestimated LOP by 13%, while Smart Cuffs and Suji underestimated LOP by 15% and 13%, respectively, compared to Zimmer. BPPO displayed comparable performance to Zimmer. Furthermore, algorithm-based LOP estimations demonstrated a significant underestimation by 24% [Tuncali et al. (19)] and a significant overestimation by 12% [Loenneke et al. (20)]. These observed discrepancies in LOP measurements between BFR devices may impact the accuracy of pressure application during BFR training and rehabilitation depending on the chosen device. Collectively, these findings emphasize the need for practitioners to carefully consider device selection, measurement posture, and the limitations of algorithm-based estimations when prescribing BFR exercise.

The findings of this study underscore the significant impact of both device selection and posture on the measurement of LOP. The variability in LOP values observed between devices aligns with previous research (13), emphasizing the inconsistency and lack of standardization across commercially available BFR systems. Moreover, posture was shown to influence LOP measurements, with values in the supine position consistently lower than those obtained when standing, which is again similar to previous research (14). Inaccurate LOP readings can have significant consequences: overestimation may lead to unnecessarily high cuff pressures during BFR exercise, potentially increasing acute fatigue (28), reducing exercise tolerance (29), and may reduce long-term adherence to an exercise programme; underestimation may yield insufficient restriction, diminishing the effectiveness of BFR training. Among the devices tested, BPPO in the supine position and Smart Cuffs in the standing position were most closely related to the gold-standard Zimmer tourniquet, suggesting their suitability as reliable, low-cost alternatives. Notably, BPPO performance is consistent with the findings of Brekke et al. (30). While Suji was the only device to demonstrate acceptable agreement through a minimal bias in comparison with the Zimmer in the supine position, most devices in both postures demonstrated issues with acceptability through bias, wide limits of agreement, or poor correlation data (31). Interestingly, LOP measured with AirBands was consistently different from that measured with the Zimmer, suggesting lower validity. This contrasts with the findings of Zhang et al. (31), who reported good validity, along with excellent test–retest reliability in measuring arterial occlusion pressure in the lower limb which might be due to likely difference in the populations. However, Keller et al. (18) have reported that AirBands were limited to a maximum of 270 mmHg for LOP measurement (18, 31). In contrast, following the recent software update, we observed measurements exceeding this threshold, with approximately 57% of our values surpassing 270 mmHg. Notably, no device demonstrated accuracy of LOP measures comparable to the Zimmer across both postures, reinforcing that LOP measurements are context-sensitive and cannot be generalized without accounting for body position. These findings affirm previous evidence [e.g., Loenneke et al. (20)] supporting the use of algorithm-based or blood pressure-derived LOP estimates in resource-limited environments (20). Practitioners should avoid assuming that a given LOP setting is interchangeable across devices or body positions. Instead, individualized LOP assessment, validated against a known standard, should be considered best practice to ensure safety and efficacy in BFR training applications. For practitioners and researchers, this study provides guidance: prioritize devices like BPPO or Smart Cuffs when affordability and accuracy are both critical, and be cautious with devices such as AirBands, especially when precise occlusion pressure is essential. The variability caused by device dependency and position differences poses a significant challenge for clinical application and research, where accurate and reliable pressure determination is recommended for ensuring safety and efficacy.

Variability also existed between devices for ROD. In the supine position in comparison to the Zimmer device, AirBands, Smart Cuffs, BPPO, and Suji produced greater discomfort by approximately 6%, 23%, 36%, and 63%, respectively. In the standing position, discomfort was 6% lower with AirBands, while Smart Cuffs, BPPO, and Suji continued to produce more discomfort by 13%, 18%, and 34%, respectively, relative to Zimmer. Given the discrepancies in LOP measurements, it also appears that ROD alone does not provide an accurate representation of the pressure-discomfort relationship. Therefore, the LOP.ROD^−1^ ratio was examined in an attempt to capture the interplay between applied pressure and perceived discomfort. The LOP.ROD^−1^ ratio revealed that BPPO, Suji, and Smart Cuffs had significantly lower values than the Zimmer device in both supine and standing position, indicating greater discomfort at lower occlusion pressures, which persisted across positions. Interestingly, the LOP.ROD^−1^ ratio for AirBands against Zimmer was statistically similar and so despite differences between these two devices for LOP and ROD, the relative discomfort with changing pressure did not appear to change (Supplementary Figure S5). For practitioners, these findings highlight the importance of considering user comfort when selecting BFR cuffs. BPPO, Suji, and Smart Cuffs may lead to higher perceived discomfort even at lower pressures, which could affect participant tolerance and, therefore, training experience/outcomes. While AirBands showed comparable comfort to Zimmer, the lack of statistical significance warrants cautious interpretation given the apparent differences in the determination of LOP. Ultimately, cuff selection should balance both pressure accuracy and user comfort, especially in populations sensitive to pressure or new to BFR training.

One limitation for this study was that LOP values for the Zimmer and AirBands devices were not able to be measured by the same pressure sensor that was incorporated into the BPPO, Smart Cuffs and Suji device systems, and so measures with Zimmer and AirBands relied upon the data displayed by these two devices, which may affect data accuracy. In addition, the blood pressure cuff used in the BPPO system had a wider cuff width compared to the other devices. Given previous studies have suggested that wider cuffs require a lower pressure to achieve LOP, similar cuff widths of the BPPO system may yield higher LOP (32–34). Therefore, the wider cuff of the BPPO system may have influenced LOP values in our study, and this methodological difference should be considered when interpreting the results and comparing across devices. It is worth noting that when using standard BP cuffs for BFR training, including the determination of LOP, participants with larger thigh girth often require longer cuffs, which are also wider, potentially influencing the pressure applied and complicating standardized measurements. Lastly, the study sample consisted of young, recreationally active males, which limits the generalizability of the findings to other populations such as females, older adults, or less active populations.

## Conclusion

This study highlights the significant variability in LOP measurements across different BFR devices and the influence of body position. When compared to the surgical-grade Zimmer tourniquet, no device demonstrated consistent accuracy across both supine and standing positions. BPPO aligned most closely with Zimmer in the supine position, while Smart Cuffs showed better agreement in the standing position. In contrast, AirBands exhibited the greatest deviation from Zimmer but were associated with comparatively greater comfort, suggesting potential suitability for individuals more sensitive to pressure. These findings underscore the need for caution when generalizing LOP values between devices or assuming their interchangeability between different positions/postures. From a practical perspective, these findings emphasize that device variability is not merely a technical concern but can have direct implications for patient safety, exercise prescription, and training outcomes for rehabilitation, health or performance. Inaccurate or non-standardized LOP values may lead to inappropriate loading, reduced efficacy, or heightened risk of adverse responses. Therefore, clinicians and practitioners are encouraged to avoid assuming device interchangeability and instead conduct individualized LOP assessments, and if possible to contextualise against a validated gold standard. This approach will help to support an appropriate BFR pressure selection and user experience to support BFR applications that are effective and well-tolerated.

## Data Availability

The raw data supporting the conclusions of this article will be made available by the authors, without undue reservation.
