# Supplementary material for: A comparison of blood flow restriction devices to assess limb occlusion pressure in supine and standing positions
Source: Front Sports Act Living. 2025 Nov 4;7:1654522. doi: 10.3389/fspor.2025.1654522 (PMC12623364; doi:10.3389/fspor.2025.1654522)
Supplement: Supplementary file 2 [file Datasheet2.pdf]

Table 1b: *Pearson correlation coefficients (r) among LOP measured in the standing position using five different devices: Zimmer, BPPO, AirBands, Smart Cuffs, and Suji. Degree of freedom is 19. Bold values indicate statistically significant correlations (p < 0.05). All correlations are two-tailed.*

		**Zimmer**	**Smart Cuffs**	**Loenneke et al.** (2015)	**Tuncali et al.** (2006)	**AirBands**	**Suji**	**BPPO**
**Zimmer**	**Pearson's r**	—						
	**p-value**	—						
**Smart Cuffs**	**Pearson's r**	0.771	—					
	**p-value**	**<.001**	—					
**Loenneke et al.** (2015)	**Pearson's r**	0.575	0.668	—				
	**p-value**	**0.006**	<.001	—				
**Tuncali et al.** (2006)	**Pearson's r**	0.412	0.501	0.846	—			
	**p-value**	0.063	0.021	<.001	—			
**AirBands**	**Pearson's r**	0.411	0.411	0.414	0.430	—		
	**p-value**	0.064	0.064	0.062	0.052	—		
**Suji**	**Pearson's r**	0.295	0.415	0.222	0.311	0.404	—	
	**p-value**	0.195	0.061	0.334	0.171	0.069	—	
**BPPO**	**Pearson's r**	0.317	0.325	0.507	0.330	0.105	0.149	—
	**p-value**	0.162	0.150	0.019	0.145	0.651	0.519	—
