# Supplementary material for: A comparison of blood flow restriction devices to assess limb occlusion pressure in supine and standing positions
Source: Front Sports Act Living. 2025 Nov 4;7:1654522. doi: 10.3389/fspor.2025.1654522 (PMC12623364; doi:10.3389/fspor.2025.1654522)
Supplement: Supplementary file 3 [file Datasheet3.pdf]

Table 1a: *Pearson correlation coefficients (r) among LOP measured in the supine position using five different devices: Zimmer, BPPO, AirBands, Smart Cuffs, and Suji. Degree of freedom is 19. Bold values indicate statistically significant correlations (p < 0.05). All correlations are two-tailed.*

		**Zimmer**	**BPPO**	**AirBands**	**Smart Cuffs**	**Suji**
**Zimmer**	**Pearson's r**	—				
	**p-value**	—				
**BPPO**	**Pearson's r**	0.636	—			
	**p-value**	**0.002**	—			
**AirBands**	**Pearson's r**	0.547	0.100	—		
	**p-value**	**0.010**	0.666	—		
**Smart Cuffs**	**Pearson's r**	-0.115	0.043	-0.408	—	
	**p-value**	0.619	0.853	0.066	—	
**Suji**	**Pearson's r**	-0.435	-0.748	-0.003	-0.089	—
	**p-value**	**0.049**	<.001	0.991	0.702	—
