# Supplementary material for: A comparison of blood flow restriction devices to assess limb occlusion pressure in supine and standing positions
Source: Front Sports Act Living. 2025 Nov 4;7:1654522. doi: 10.3389/fspor.2025.1654522 (PMC12623364; doi:10.3389/fspor.2025.1654522)
Supplement: Supplementary file 4 [file Datasheet4.pdf]

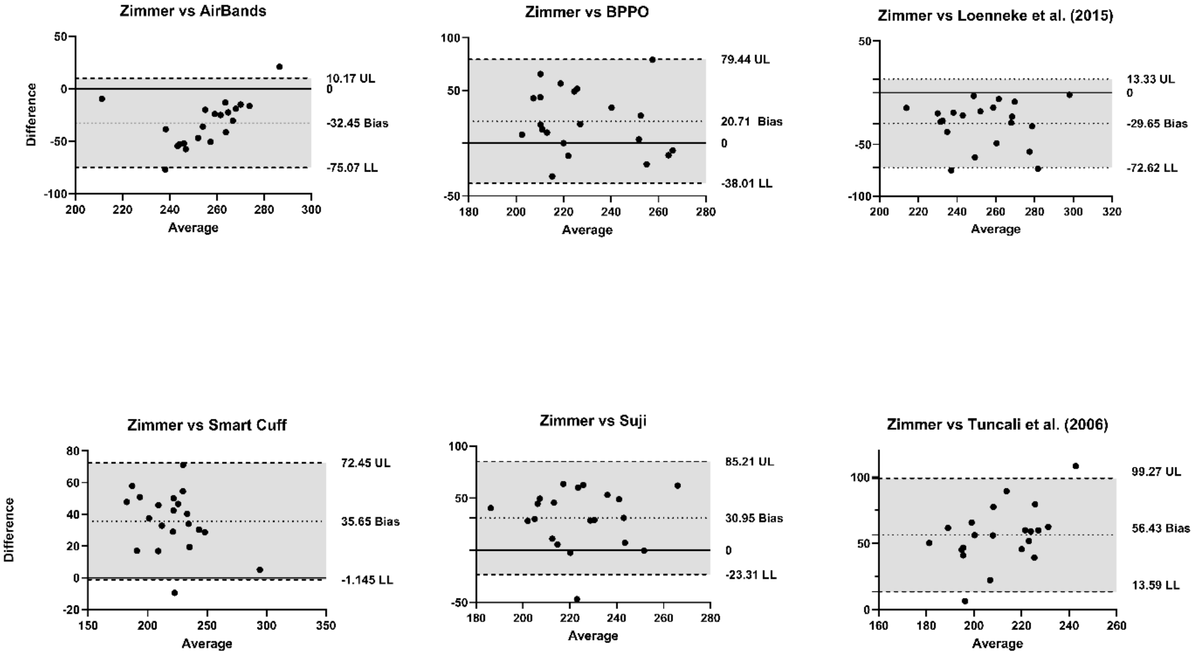

Supplementary Figure 2: Bland–Altman plots illustrating the agreement between limb occlusion pressure (LOP) measurements obtained in the standing position from four commercially available BFR devices (AirBands, BPPO, Smart Cuffs, and Suji) and two algorithm-based LOP compared to the surgical-grade Zimmer device. The solid black dashed lines represent the limits of agreement (±1.96 SD) with their corresponding 95% confidence intervals shown as shaded areas. The mean bias is indicated by a dotted black line, also accompanied by its 95% confidence interval.
